# Women's perspectives on illness when being screened for cervical cancer

**DOI:** 10.3402/ijch.v72i0.21089

**Published:** 2013-08-05

**Authors:** Lise Hounsgaard, Mikaela Augustussen, Helle Møller, Stephen K. Bradley, Suzanne Møller

**Affiliations:** 1 Institute of Nursing, and Health Science, University of Greenland, Nuuk, Greenland; 2 Research Unit of Nursing, University of Southern Denmark, Odense, Denmark; 3 Ministry of Health and Infrastructure, Greenland; 4 Department of Health Sciences, Lakehead University, Thunder Bay, ON, Canada; 5 School of Nursing and Midwifery, National University of Ireland, Galway, Ireland; 6 Institute of Nursing and Health Sciences, University of Greenland, Nuuk, Greenland

**Keywords:** cervical cancer, HPV, Greenland, interview, nursing, perceptions of health and disease, public health programming, screening

## Abstract

**Background:**

In Greenland, the incidence of cervical cancer caused by human papillomavirus (HPV) is 25 per 100,000 women; 2.5 times the Danish rate. In Greenland, the disease is most frequent among women aged 30–40. Systematic screening can identify women with cervical cell changes, which if untreated may cause cervical cancer. In 2007, less than 40% of eligible women in Greenland participated in screening.

**Objective:**

To examine Greenlandic women's perception of disease, their understanding of the connection between HPV and cervical cancer, and the knowledge that they deem necessary to decide whether to participate in cervical cancer screening.

**Study design:**

The methods used to perform this research were 2 focus-group interviews with 5 Danish-speaking women and 2 individual interviews with Greenlandic-speaking women. The analysis involved a phenomenological-hermeneutic approach with 3 levels of analysis: naive reading, structural analysis and critical interpretation.

**Results:**

These revealed that women were unprepared for screening results showing cervical cell changes, since they had no symptoms. When diagnosed, participants believed that they had early-stage cancer, leading to feelings of vulnerability and an increased need to care for themselves. Later on, an understanding of HPV as the basis for diagnosis and the realization that disease might not be accompanied by symptoms developed. The outcome for participants was a life experience, which they used to encourage others to participate in screening and to suggest ways that information about screening and HPV might reach a wider Greenlandic population.

**Conclusion:**

Women living through the process of cervical disease, treatment and follow-up develop knowledge about HPV, cervical cell changes, cervical disease and their connection, which, if used to inform cervical screening programmes, will improve the quality of information about HPV, cervical cancer and screening participation. This includes that verbal and written information given at the point of screening and diagnosis needs to be complemented by visual imagery.

Drawing on the reported experiences of Greenlandic women who have been treated for cervical cell changes, this research identifies the knowledge which women view as necessary to understand the connection between human papillomavirus (HPV), disease and the importance of cervical cancer screening participation.

Out of a population in Greenland of 56,749 in 2012, 26,208 were women. The population is young, with 40% of women below 25 years; with approximately 19% of the population residing in small villages in rural and remote areas. Accessibility is a particular challenge, especially in winter with sea ice (1).

The incidence of cervical cancer in Greenland is among the highest in the world with 25 per 100,000 women (2); with the majority of the cases occurring among women aged between 30 and 40 (3), which is about 5–10 years earlier than in Denmark. The introduction of systematic screening has been shown to reduce the risk of women developing cancer by 80% (4). The establishment in 1998 of a nationally organised screening programme in Greenland for women between the ages of 18 and 65 did not result in a satisfying rate of participation – only 40% in 2007 (3); while the European Council recommends 85% participation (5). The screening programme in Greenland aims to reach women aged between 18 and 65. Retrieved from a central population registry, women are mailed a written invitation to partake in screening, in the form of a pap test at their local health centre or hospital, performed by a nurse or doctor (3).

Cervical cancer is the third most common cancer among women globally (6). The incidence in Greenland is, as noted, 25:100,000; 2.5 times the Danish rate. Among Canadian Inuit, the incidence is 14.7, about 3 times the Canadian incidence (7).

Systematic screening with smear tests can identify cell changes in the cervix, which, left untreated, may lead to cervical cancer in about 5–10 years. The resulting survival is more than 99% at 5 years when abnormal cells are removed. Currently, it is possible in Greenland, at 12 years of age, to vaccinate for the variations of HPV that are most likely to cause cancer (6). For most women in Greenland, this is prior to first sexual activity (8).

Research indicates, though, that the vaccine is not 100% effective; thus, it is important that even women who have received the vaccination partake in regular screening (9). In addition, most women aged between 20 and 65 have not been vaccinated and may already be infected with HPV. Participating in regular screening would be to their great advantage. The low rate of participation in screening in Greenland may have several reasons. One is that only 50% of possible participants were invited to partake due to software issues that existed many years. Another is that women may choose not to partake despite having been invited (3).

In Danish studies, some women reported not participating in screening because they felt healthy and therefore did not think they would have cervical cell changes (10, 11). Others did not participate because they could not cope up with the idea of possible disease due to family obligations or a demanding job (10). Some Danish and Canadian First Nations women did not participate because of being embarrassed about, and uncomfortable with, the procedure and/or because they did not feel they had the knowledge required to participate – with Danish women thinking they would be screened for manifest cancer, which they did not need as they felt healthy (11, 12). A British research team reported similar findings in a study involving 600 women who were screened in a public sexual health clinic. Seventy one percent of participants thought they were being examined for having manifest cancer, while only 10% knew that they were part of a preventive screening programme (13).

Danish women and Canadian First Nations women prefer to receive information about sexual health and cervical screening from one-to-one conversations with a health care provider (11, 14). This research extends the previous research by focusing on Greenlandic women's understanding and preferences to improve screening programmes and participation among Greenlandic women.

The objective of this research was to examine women's perception of disease, their understanding of the connection between HPV and cervical cancer when they have cell changes and what knowledge they deem necessary for tri-annual cervical cancer screening participation. In addition, this knowledge, along with information about the vaccine itself, may help parents to make a more informed decision about whether to vaccinate their children for HPV at 12 years of age.

## Design

### Interviews

In February 2012, 2 focus-group interviews were conducted with a total of 5 Danish-speaking women aged 30 to 45 living in the capital Nuuk. One group had 2 participants and one group had 3. The women had had a cone surgery between a few days and 5 years previously, and were in a follow-up process. Two individual interviews were conducted with Greenlandic-speaking women aged 22 and 23 living in 2 different villages. They had recently been diagnosed with abnormal cervical cells to be treated and were admitted for surgery at the gynaecological ward at the Queen Ingrid Hospital in Nuuk. Because of their short stay in Nuuk, it was not possible to conduct focus-group interviews. All the participants were recruited via letters of invitation, through the gynaecological ward at the Queen Ingrid Hospital.

The focus-group interviews lasted 1 and 1.5 hours and were conducted in Danish by Lise Hounsgaard, Mikaela Augustussen and Suzanne Møller. The individual interviews lasted an hour each and were conducted in Greenlandic by Mikaela Augustussen. For both group and individual interviews, a semi-structured interview guide stimulated dialogue. The guide explored issues around: what it means to be diagnosed with cervical cell changes; understanding of cervical cell changes and the connection between HPV and cervical cancer; how knowledge about cervical cell changes is sought and, what knowledge is necessary for women to agree to participate in screening. The sample of participants discussed experiences regarding their diagnosis of cervical cell changes, treatment and follow-up.

### Analysis

Each interview was transcribed verbatim as one comprehensive text in preparation for interpretation. A phenomenological-hermeneutic approach informed by the French Philosopher Paul Ricoeur (15) was used. Ricoeur views interpretation as a dialectic movement between parts of the text and the text as a whole and between explanation and understanding. Understanding the text requires following it from the manifest description (what the text conveys) to what it talks about (15). This method has been adjusted for nursing research by Scandinavian nursing researchers (16–18). The analysis involves 3 analytical levels, naive reading, structural analysis and critical interpretation (15).

The first step in the analysis, the naive reading, involved a “non-judgmental” reading of the text, which opened up for insights into the meaning of the text as a whole. The structural analysis was subsequently carried out to explain what the text talked about. As demonstrated in Table I, this analytical step involved movement from units of meaning to units of significance. The analysis was completed with a critical interpretation and discussion.

**Table I:** Three levels of analysis: units of meaning, units of significance and central themes

Units of meaning *What is being said* *(non-judgmental reading)*	Units of significance *What is being talked about* *(Structural analysis – primary interpretation)*	Central themes *(critical interpretation)*
*I thought I was going to die when the doctor called me at home and told me* *I feel vulnerable when I think about the fact that something happens to me that I have no control over* *It is strange to have something like that thrown at you, that you are sick, when you do not feel sick at all. You know? …*	*The meaning of cervical cell changes* *Perceptions of the body* *Knowledge about HPV*	*Potential disease*
*My husband takes more care of me. I can feel that. Although he does not say much* *“My oldest daughter lives in another town; she came home [when I told her I had [cervical]cell-changes]”*	*Anxiety about loss*	*Vulnerability*
*“I have found out that my mom has had it. She has been ‘scraped’ twice. That scared me quite a bit”* *[I will think about it ] if there is something on TV about cancer or something about [cervical] cell-changes … It does not affect me much in my daily life – operated 5 years ago*	*Break in the family history* *A new identity*	*Disease as life-experience*
*I have not heard about anyone being called. Basically people have just gone on their own initiative … when we talk about it and I realise people have not had a smear I ask them to go and get it done*	*The connection between HPV and cell changes* *Ideas for initiatives*	*New authoritative competence to act*

### Ethical considerations

The study was conducted according to the ethical guidelines for nursing in the Nordic countries (19). Ethical approval was obtained from the Research Ethics Committee for Health Research in Greenland (No. 2011-057604) and written informed consent was obtained from all participants.

## Results

The non-judgmental reading indicated that the participants did not understand the meaning of the diagnosis, that they were anxious about the diagnosis, that they did not have any symptoms to “support” the diagnosis and that they lacked knowledge about HPV. With the structural analysis, 4 themes were identified; potential illness, vulnerability, illness as life experience and authoritative competence to act. The 4 themes are critically interpreted and discussed below.

### Potential disease

Some participants received the information that their cervical screening result showed cell changes and potential disease through letter and others through a phone call. The mode of information through which the result was given was significant for the way it was perceived. One woman said: “One night I was called by the doctor at home; she said that I had cell-changes. The doctor has never called me at home before so I was totally floored”. The woman's thought was that cervical cell changes had to be very serious since the doctor called at night after business hours. It made her think she was suffering from something very serious. Another woman had a similar anxiety-provoking experience. She recalled “the doctor said that cell-changes may be a precancerous stage – I don't really remember – My partner was with me the whole way; he said that I went totally blank …”.

The differentiation that health-care providers make between cervical cell changes and precancerous cells cannot be easily explained to the women who have been screened. At the point of diagnosis, the women have difficulty in understanding cervical cell changes as anything but disease with early-stage cancer, which is very anxiety provoking. The women feel that healthy lifestyles, exercise regimens and healthy body weights contribute to them being as healthy as they feel. That it may not be so, is confusing. A young woman with a healthy body weight sought the advice of her boyfriend who is a dietician about whether her diet contributed to her developing cervical cell changes. She said: “I smoke five cigarettes a day, I do not exercise that much but I eat a varied diet. Is it my own fault? Have I not done enough? You know, where can I put the blame?”. This participant did not consider smoking to be a risk factor and was not informed by her dietician boyfriend that it is. Generally, the women perceive themselves to be healthy which contributes to them not expecting their screening to show disease in the form of cervical cell changes. Another woman said: “I felt nothing. It is strange to have something like that thrown at you, that you are sick, when you do not feel sick at all”.

Augustussen (20) reports similar findings in a Greenlandic context where women participants felt insecure about the level of their own health, when they could not be sure that no bodily symptoms was equivalent to not being sick. One woman in this study explained: “I think it has to do with it being on the inside. I cannot see it in the same way I can a cut on my hand, which I can observe healing … or not”. This woman and several others appear to express a sort of alienation when she calls the cervical cell changes “it”. This phenomenon has previously been described. Forss et al. introduced the concepts of “objectification” and “subjectification” in a Swedish study of screened women (21, 22). They write that with “subjectification” the information about cervical cell changes is internalized and the cells become part of the woman herself. With “objectification”, the cells become an “it”; an object lying outside the woman's body, the interpretation of which often requires the help of a health-care practitioner. The process of internalization is made more difficult due to the lack of symptoms.

One way to help women internalize was examined in an Australian study in 1997. The women who participated in the study had the opportunity to see what the doctor viewed during a colposcopy. The visual experience made it possible for them to reconcile the presence of the cells in their bodies (23). Including a visual representation of what is seen in the colposcopy would help women to develop a more subjective understanding of the disease, connections between HPV and cervical disease, and the importance of screening participation.

Only one of the interviewed women had knowledge about the connection between HPV, cervical cell changes and cervical disease. Another said: “I've heard all sorts of things like, you will get it [cervical cancer] if you have early sex, or if you get sexually transmitted diseases and all sorts of other things, but whether it is true or not, I do not know”. One woman who lives in a remote village and speaks only Greenlandic noted: “I searched the net but did not find anything”. Generally, the accessibility of information for women, who live in a village, is different than for the women who live in Nuuk (20). It appears that among the women interviewed those who live in remote settings, search the Internet less often, and those who do may not find anything in their mother tongue Greenlandic. In addition, they often do not have the opportunity to speak with a health professional until after they have been informed about the cervical cell changes and are in Nuuk for treatment. The interviewed bilingual Greenlanders were able to access information in Danish. They were not, however, satisfied with the quality of the information they found. An Australian study shows that misinformation may result when people search the Internet for information without some guidance from a health-care provider about appropriate websites (24). As noted in a study (12), several participants felt ashamed that they had been infected with HPV because websites emphasised that HPV is a sexually transmitted disease.

To summarise, participants noted a lack of knowledge about what HPV means in relation to cervical cell changes, what cervical cell changes mean in relation to being treated for it and a lack of ability to find quality information in one's mother tongue. This points to the importance of the developing information material in both Greenlandic and Danish which is easily accessible on the Internet, or is given as a pamphlet when women are screened as suggested by several participants.

### Vulnerability

Participants expressed that they had felt vulnerable. This vulnerability resulted from the information that they have cervical cell changes; as a woman recounts: “When I was told I had cell-changes I was very confused. Did this mean I had cancer, was it all just terrible? I cried and cried …”. Any prior experience the women had had with disease revolved around common childhood diseases, throat infections, the flu, and the common cold; all of which exhibit external bodily symptoms with rashes, fever, pain somewhere in the body, a runny nose and so on.

Lack of knowledge about what the screening results mean, along with having no symptoms, may be what increases anxiety and feeds the imagination that cervical cell changes may have fatal consequences. These thoughts were expressed in a variety of ways, although generally focussed on cancer and impending death; for example “I may not see my son grow up”.

The nursing theorist Travelbee discusses the possibility that the origin of anxiety comes from our ability as human beings to imagine our future and have expectations about how it will unfold (25). In the context of this study, it appears that an outcome of the screening result is that the women are suddenly forced to contemplate their own mortality. The fear of the cervical cell changes being equivalent to cancer may create a general human anxiety about death and the realization that life is not limitless (17).

Similarly, it appears that feelings of vulnerability are also intensified among the women's close relatives. This is expressed in the need to care for and be with the screened women in their role as mother and spouse. As noted by a woman: “My oldest daughter lives in another town; she came home [when I told her I had cervical cell-changes]. All of a sudden she wanted to come home, and my husband takes more care of me. I can feel that”.

In summary, the women noted that potential disease heightened feelings of vulnerability, not only in themselves, but also in their close relatives. This vulnerability appeared to be caused in part due to lack of knowledge or misinformation about the connection between cervical cell changes and cervical cancer; seeming to occur when humans are feared of losing something or somebody they feel close to, or feel that loss is an increased threat (25).

### Disease as life experience

The screening result represents a break in everyday life in relation to family, friends and colleagues. The moment when the screening result was received, participants experienced anxiety and fear of death due to cancer. Hounsgaard found similar reactions among women with cervical cell changes in Denmark (17). To overcome the anxiety, caused by the fear of cancer, they had to live through the fear and acknowledge it as an expression of suffering. Living through the fear is a reflexive process in which the screened women re-interpret the disease history of their families. This process and the company of their close family members allows them to also recount their own disease history, which is created and recreated to eventually become a coherent history that is meaningful within the family disease history. As a woman expressed it: “I have found out that my mom has had it. She has been 'scraped’ twice. That scared me”.

The task of recreating meaning in their everyday lives, after being diagnosed with cervical cell changes, occurs through a re-conceptualizing of the family disease history and the women placing their own disease history within the frame of the family history. Both disease histories develop a new dimension. The women's own experiences become an active part of the family's disease history, which can be retrieved if other members of the family become sick (17).

This new narrative also comes into effect in relation to close friends, as recounted by a woman: “You are at an event having coffee, for example … A bunch of women gathered together and the conversation always turn to … perhaps someone has just had a smear done and someone asks ‘have you had such and such done?’”. The woman identifies that when the conversation involves the pelvic area, she can contribute with experiences from her own narrative and contact with the health-care system.

In summary, the new knowledge and experiences may cause the woman's everyday life to be different from before she lived through the fear of dying in the process of cervical cell changes. In addition, increased knowledge and experience appears to involve that women develop a new identity narrative and a new view on their family disease history which in turn creates a “new authority”, as described below.

### New authoritative competence to act

“It is just like a general health check. It just has to be done”. Through reflecting on the process of cervical cell-changes, the women's understanding of disease evolves (20). From not knowing that symptoms do not have to be present to make the diagnosis of cervical cell changes, the women develop the understanding that screening is a way to detect precancerous stages before they develop into cancer. As noted by a woman 5 years past her cone surgery: “I think everyone ought to be screened, everyone I know” (14). Understanding the connection between HPV and cervical cell changes also led the women to advocate for a vaccination being given, especially to younger women before they engage in sexual activity, and in an interest in having their own children vaccinated. In addition, their experiences with potential disease led to suggestions about possible interventions, which the women felt might improve screening participation (see Table II).

**Table II:** Interviewed women's suggestions for possible interventions to improve screening

*You could present something about it on the radio. Many women listen to the radio… they need to know what may happen, what may develop.* *We also need to include it in the sex education in the schools.* *More information through the media – television, radio, the newspapers through the workplaces the cantinas.* *I would like a pamphlet. What may happen, what happens and all that. A bit of drawing showing where they operate. How much is removed. Just a little bit.*

The women stress the importance of developing strategies, which focus on increasing motivation to participate, and consider the women who choose not to participate in screening. In a study on barriers to screening participation carried out by the Danish Cancer Society, focus group interviews with women identified several barriers; including that some women made a conscious choice not to participate often due to erroneous or limited knowledge, because they felt a lack of personal relevance or did not feel that anything was wrong, that they were afraid of a cancer diagnosis or what cervical cell changes would mean, or that they were afraid of the examination being uncomfortable. Others did not get the invitation to participate because of having moved, or they had forgotten to make an appointment with the General practitioner (GP) after receiving an invitation, or they had not kept their appointment and this was not followed up by the GP (11). Several studies show that the general practitioner plays an important role in removing these barriers (12, 14, 26, 27). Much international research (6, 28) demonstrates that a targeted focus on lack of participation may increase participation significantly, particularly if a combination of initiatives are utilised. One participant raises another important issue, when she discusses about systematic screening invitations or a lack thereof. She said: “I have not heard about anyone being called in for a smear. It has generally just been women who have gone on their own initiative” and continued that this initiative was sparked by herself encouraging them to do so.

In summary, while women who have undergone treatment for cell changes serve as educators in their social circles, a well-organised screening programme, in addition to a wide distribution of information and educational opportunities both one-to-one with a health-care provider and in the form of mass media campaigns, work place sessions and sex education in the schools, is a prerequisite if the goal is to increase screening participation rates. This has also been emphasised by WHO (6) and by Arbyn and colleagues (5) discussing the challenges of organizing screening programmes in the 15 old member states of the European Union.

### Limitations

In a phenomenological-hermeneutic interpretation, one can never be free of one's pre-understanding. As social scientists and researchers, we have to engage in critical reflection (29). We are aware that the interpretation in this study represents only 1 possible interpretation. Also, 2 interviews were translated from Greenlandic to Danish and then to English, which may cause meaning to be lost.

## Conclusion

This is the first published study to attempt to describe women's awareness, attitudes and experiences of cervical cancer, screening participation and HPV vaccine in a Greenlandic population.

Findings include that, as do women elsewhere, Greenlandic women experience potential disease with strong feelings of anxiety about their health and future, and with feelings of alienation towards the diagnosis, which they objectify rather than internalise. These feelings might be due to lack of knowledge about HPV and cervical disease, the connection between the 2 and screening, which also appear to be connected to low rates of participation. These Greenlandic women believe that a prerequisite for increased participation rates and mitigation of anxiety and alienation is increased levels of knowledge. Their suggestions also represent the need to have a well-functioning nationally organized screening programme.

Our recommendations for clinical practice, based on women's lack of knowledge about smears and HPV and due to a high turnover of health professionals in small towns and villages, are to focus on further education about cervical cancer and its prevention. Some of the information could be given through improved one-to-one conversation with a health-care provider. However, increased public information campaigns in the form of pamphlets, posters, audio or audiovisual advertisements must be offered in addition to sex education in schools and workplace educational sessions. Such initiatives may help to increase public awareness and thus participation in screening, and would increase the quality of health promotion and illness prevention, but a prerequisite for them to be successful is that the National Screening program runs smoothly.

Future research should focus on determining if there are certain subsets of the female population who are under-screened for cervical cancer and how screening coverage can be increased within these groups. In addition, it may be beneficial to examine whether knowledge, awareness and cervical screening participation rates would increase if women are mailed self-examination kits for HPV along with an information sheet about HPV, cell changes, and cervical cancer and their connection. This initiative is being examined in North-western Ontario currently and has been examined with positive results for increased screening among Inuit women in Nunavik, Canada (14, 30).
